# Equine echocardiography: Can dobutamine infusion correct alterations due to sedation with alpha-2 agonists?

**DOI:** 10.1371/journal.pone.0276256

**Published:** 2022-10-18

**Authors:** Valentina Vitale, Tommaso Vezzosi, Chiara Di Franco, Angela Briganti, Rosalba Tognetti, Giuseppe Conte, Elena Bucchioni, Micaela Sgorbini

**Affiliations:** 1 Department of Veterinary Sciences, University of Pisa, San Piero a Grado, Pisa, Italy; 2 Department of Agricultural, Food and Agro-Environmental Sciences, Pisa, Italy; 3 Private Practitioner, Pisa, Italy; Universidade Federal de Minas Gerais, BRAZIL

## Abstract

For the echocardiographic examination horses should not be sedated unless absolutely necessary because this alters cardiac dimensions and indices of function. However, some horses do not tolerate the echocardiographic procedure and require sedation to conduct the examination safely and obtain good quality images. The objective of this study was to evaluate whether the concurrent infusion of dobutamine in horses sedated with romifidine counteracts the cardiovascular changes observed with sedation alone. Twelve healthy untrained Standardbred mares were used. Three echocardiographic examinations were performed on the same day for each subject: a) without any treatment under resting conditions (WT); b) under sedation with romifidine administered intravenously (RT); c) under sedation with romifidine and concurrent intravenous infusion with dobutamine (RDT). A three-hour washout period was observed between each examination and the order of the examinations was randomly decided by rolling a dice. The measurements on the images recorded were performed offline at the end of the study protocol and at this point the operator was blinded to the horse and treatment administered. Left ventricular internal diameter (LVID) in diastole, left ventricular free wall (LVFW) in systole, and fractional shortening (FS) were higher in the WT group compared with the other two groups. No differences in the other M-mode and B-mode values were observed. A continuous rate infusion of dobutamine did not counteract the alterations caused by sedation and led to similar echocardiographic measurements to those obtained after romifidine administration.

## 1. Introduction

Echocardiography is part of every comprehensive cardiac examination in horses [1]. Cardiac ultrasound is recommended in several scenarios, such as auscultation of a cardiac murmur, detection of arrhythmias, muffled heart sounds, in the setting of poor performance when musculoskeletal and respiratory diseases have been ruled out, in horses with unexplained pyrexia, and when there are signs of congestive heart failure [1–3].

For the echocardiographic examination horses should not be sedated unless absolutely necessary because this alters some cardiac dimensions and indices of function [2]. Alpha-2 agonists primarily affect M-mode measurement of the left ventricle (LV) and aorta (AO) in systole [4].

Previous studies have demonstrated that detomidine, and to a lesser extent romifidine, influences heart function and dimensions with an increase in left ventricular internal diameter (LVID) in systole and a reduction in both interventricular septum (IVS) in systole and fractional shortening (FS%) [4,5]. Furthermore, a slight increase in the frequency of aortic and pulmonary regurgitations has been observed under sedation [4]. Acepromazine appears to have less effect on the echocardiographic parameters, but it has been shown to increase the diameter of the pulmonary artery (PA), AO and IVS and to decrease the diameter of the left atrium in diastole [2,4,6]. However, some horses do not tolerate the echocardiographic procedure and require sedation to conduct the examination safely and to obtain good quality images [1].

Dobutamine infusion has been used alone or in combination with atropine for pharmacological stress tests in humans and horses [7–13]. Recommendations for stress echocardiography in horses include examinations of patients with mild to moderate heart valve insufficiencies, with mild to moderate myocardial hypertrophy or dilation, as well as to detect subclinical cardiovascular abnormalities that are not present at rest [8,9]. To prevent the rapid heart rate decline post-exercise, pharmacological stress protocols have been developed [14]. Infusion of dobutamine provokes an increase in LV and IVS wall thickness and a decrease in LVID both in systole and diastole [9,11].

Given the different effects on echocardiographic measurements observed during dobutamine infusion, compared to that described using alpha-2 agonists, the aim of this study was to evaluate whether the concurrent infusion of dobutamine in horses sedated with romifidine could counteract the cardiovascular changes observed with romifidine alone.

## 2. Materials and methods

### 2.1 Animals and study protocol

The study consisted in performing three echocardiographic examinations on the same day for each subject in a group of healthy horses undergoing three treatments: a) without any drugs under resting conditions (WT); b) under sedation with romifidine administered intravenously (RT); c) under sedation with romifidine and concurrent intravenous infusion with dobutamine (RDT). The order of the examinations was randomly decided by rolling a dice and there was a three-hour washout period between each treatment. The three-hour period was estimated enough, considering the half-life of the drugs used in the study. Dobutamine is a synthetic catecholamine with predominant beta-stimulation and its half-life is approximately 2 minutes [15]. Romifidine produces long-lasting sedation in horses but the decline in cardiovascular and sedative effects correlates with the decline in plasma concentration [16,17]. In particular, haemodynamic effects were observed for a maximum of 120 min following the administration [16]. During the washout period, the horses rested in a 4x4 stall box with *ad libitum* water but no hay.

With an alpha error set at 0.05 and a beta error set at 0.2, to identify a difference of 1.27 cm of the LVID in systole between the echocardiogram performed under sedation and without treatment, as described by Buhl et al, [4] a total of 13 horses needed to be enrolled.

The horses included in this study were selected from the herd owned by the–blinded for review–of the–blinded for review–. All the horses were untrained Standardbred mares housed in paddocks (75 x 75 m) consisting of ten subjects each, where they were given *ad libitum* access to both hay and water.

The study protocol was approved by the ethics committee of the Ministry of Health (Approval Number 491/2021). Horses were considered healthy based on history, physical examination, cardiac and thoracic auscultation, as well as haematological and biochemical blood work performed within one month before the study.

### 2.2. Instrumentation and monitoring

**Fig 1:**
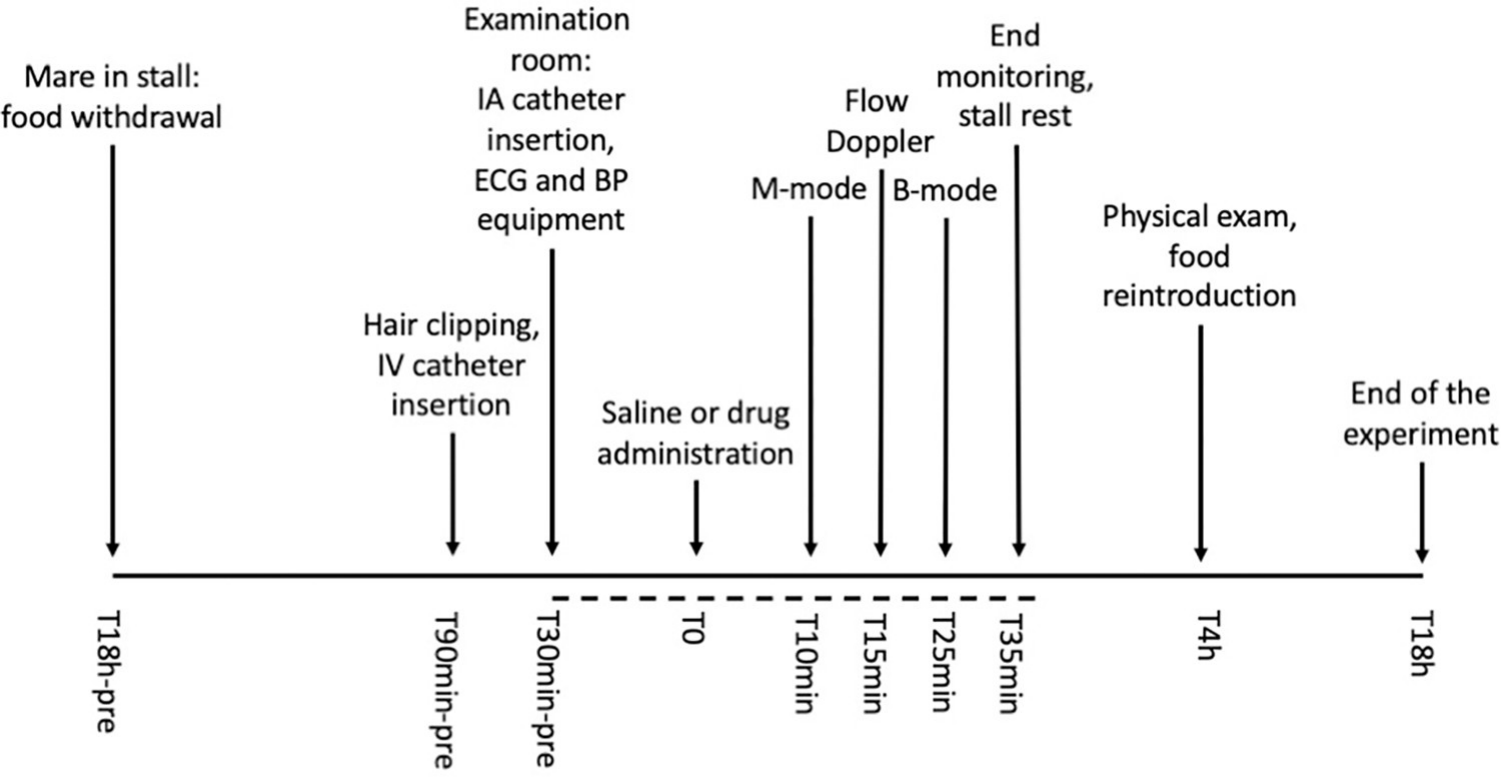
Study protocol from the day before until the end of the experiment. The dashed line indicates the part of the experiment that was repeated three times with a three-hour washout period in between. The intra-arterial catheter was maintained in place during the washout period and covered with a bandage to avoid repositioning at the next session. Four hours after the end of the entire procedure a physical exam was performed, and food was reintroduced. The following morning each mare was rechecked, and the experiment was considered concluded. IV = intravenous, IA = intraarterial, BP = blood pressure.

A detailed study protocol is reported in Fig 1.

The day before the examination, each mare was led to a 4 x 4 m stall where they had water *ad libitum*, while food was withdrawn 18 hours prior to the study (T18-pre). One hour before the protocol (T90 min-pre), a 14-gauge catheter (Surflo® IV Catheter, Terumo (Philippines) Corporation, Rome, Italy) was inserted into the left jugular vein after clipping and under aseptic conditions. The skin over the left and right transverse facial arteries was clipped and desensitized with a topical cream (Emla cream 5%, AstraZeneca, Copenhagen S, Denmark) containing lidocaine and prilocaine. Hair was clipped from 5 x 5 cm areas on the left axillary region, right jugular groove, tip of the left shoulder and cranial to the sternum, and patch electrodes were applied and fixed in place with glue. A 15 x 15 cm area was also clipped on the right axillary region, and skin was cleaned of grease and dirt using alcohol to allow optimal contact with the ultrasonographic probe.

Approximately 60 minutes later (T30 min-pre), the mare was led to the examination room, restrained in a stock, and equipped with a base-apex electrocardiography (ECG), which was recorded continuously by a telemetry system (Televet 100, Engel Engineering Service GmbH, Germany). Noninvasive blood pressure (NIBP) was monitored throughout the study with an oscillometric device (Edan Patient monitor M50, Edan Instruments Inc., Hamburg, Germany) after application of the cuff to the tail base with the bladder centred over the middle coccygeal artery. A correction factor of 10.5 mmHg was added to the recorded values. This value was obtained by multiplying the correction factor of 0.7 mmHg [18,19] by the distance from the tail base to the point of the shoulder as an estimate of the right atrial level. As all the mares were of similar height, this distance was roughly estimated to be 15 cm.

Transverse facial artery was cannulated with a 20-gauge, 45 mm catheter (Deltaven®, Deltamed S.p.a., Viadana (MN), Italy) and fixed in place with glue and adhesive tape. The catheter was flushed with heparinized isotonic saline before insertion and was connected to a pressure transducer using a 2 m noncompliant connection line. The pressure transducer was placed at the level of the point of the shoulder as an estimate for the location of the right atrium and connected to a monitor (Invasive blood pressure machine & My LabTM 30VETGold, Esaote S.p.a., Florence, Italy) with recent factory calibration. The system was zeroed to atmospheric pressure and the invasive blood pressure (IBP) was monitored continuously throughout the study [20].

According to the order of treatments, 5 mL of saline, romifidine (0.04 mg/kg) or romifidine (0.04 mg/kg) followed by continuous rate infusion of dobutamine (2 ug/kg/min) were administered through the IV catheter (T0). A single administration of romifidine, instead of a continuous rate infusion, was chosen to simulate a real-life condition in which sedation is required to restrain the patient during the examination.

Heart rate, NIBP and IBP were recorded at 0, 2, 5, 10, 15, 25 and 35 minutes after the administration of the treatment.

The ECG was monitored continuously during the whole procedure and in case of detection of HR less than 16 bpm, three or more ventricular premature beats or ventricular tachycardia the study would have been interrupted and appropriate treatment administered. Afterward the ECG recordings were examined for presence/absence of arrhythmias among the three groups and number and type of arrhythmias for each horse and treatment.

### 2.3. Echocardiography

Ten minutes after the administration of drugs or saline (T10 min), echocardiographic examination was carried out always by the same operator (VV) using an ultrasound system^g^ with a 2.5 MHz phased arrayed sector transducer with harmonic imaging. All images were recorded from the right parasternal view at a depth of 25–30 cm. Two-dimensional short-axis images of the LV were recorded as previously described [2]. The M-mode cursor was placed across the LV at the chordal level between the mitral valve and the papillary muscles, and care was taken to ensure that the plane of section was perpendicular to the IVS, and that the cursor bisected the LV.

At least five separate cardiac cycles (five frames) were recorded and stored on the ultrasound machine for subsequent evaluation and measurements. These images were obtained between 10 and 15 minutes after drug administration and used for off-line measurements of LVID, IVS and left ventricular free wall (LVFW) both in systole and in diastole. End diastolic measurements were taken just before the beginning of the contraction, while end systolic measurements were taken at the time of maximal IVS thickness.

Fractional shortening (FS%) was obtained from the LVID measurements with the following formula:

$$FS\%=\frac{LVIDd-LVIDs}{LVIDd}x\;100$$

From 15 to 25 minutes after drug or saline administration (T15 min), the mitral, aortic, tricuspid, and pulmonary valve areas were all examined in the described order using color flow Doppler, to identify regurgitant jets. The mitral and tricuspid valves were examined in the long-axis 4-chamber view. The aortic valve was examined in the long-axis LV outflow tract view. The pulmonary valve was examined in the long-axis right ventricle (RV) outflow tract view [1]. At least five 10-second clips were obtained for each valve and saved on the ultrasound machine for subsequent review.

Based on the area of the regurgitant jet in comparison with the receiving cardiac chamber, five groups were defined: no jets, very small jet (< 10% of the area of the previous chamber), small jet (10–30%), medium jet (30–50%), and large jet (> 50%) [4].

Finally, from 25 to 30 minutes after drug or saline administration (T25 min), at least five 10-second clips were obtained from: the long-axis view angled dorsally to display left atrium (LA); the long-axis view angled ventrally to display LV; the long-axis RV outflow tract view; and the short-axis aortic view. These images were stored and subsequently examined off-line to obtain the following measurements: diameter of LA in systole (right parasternal view, long axis), area and volume of LV in systole and in diastole (right parasternal view, long axis), diameter of aorta (right parasternal view, short axis), and diameter of pulmonary artery (right parasternal view in diastole, long-axis RV outflow tract). The measurements were performed as previously described [1,2]. Left ventricular volumes in systole and diastole were calculated using the Simpson method. The EF% was obtained with the following formula:

$$EF\%=\frac{LVVd-LVVs}{LVVd}x\;100$$

Stroke volume (SV) was automatically calculated from the left ventricular volume (LVV) in systole and diastole with the following formula:

SV=LVVd−LVVs

The ultrasonographer was not blinded during the procedure and the order in which the images were obtained was based on the study of Buhl et al. [4] Subsequently, the images were stored with a code that allowed to identify the horse and the treatment received and were all examined at the end of the study protocol. Measurements were performed by the same operator (VV) who was blinded to the code used to store the images. Five values were obtained from different clips or frames for each measurement, and the average was calculated and used for statistical analysis.

ECG, invasive and noninvasive blood pressure monitoring were continued for 5 minutes (T35 min), after which, the mare was disconnected from the machines and led to the stall where it was left for a three-hour wash out.

At the end of the whole experimental procedure, mares were examined and monitored for heart rate (HR) and intestinal sounds 4 hours after the last drug administration and food was reintroduced when considered appropriate (T4). After one night under observation, if the physical examination remained normal, the animals were taken back to their paddocks (T18).

### 2.4. Statistical analysis

Data were analysed for distribution using the Shapiro-Wilk test. Based on the normal or not Gaussian distribution, the results were expressed as mean (± standard error) or median (minimum, and maximum) values respectively.

Heart rate and IBP were evaluated by a two-way factorial ANOVA according to the following linear model:

y_ij_ = μ + group_i_ + time_j_ + group_i_ x time_j_ + ε_ij_

where

y_ij_ = Heart rate, systolic, mean and diastolic arterial pressures

μ = mean

group_i_ = fixed effect of the i^th^ group (WT, RT, RDT)

time_j_ = fixed effect of the j^th^ recording time (T0, T2, T5, T10, T15, T25, T35)

ε_ij_ = random error.

A chi-squared test was applied to verify the association between the degree of regurgitant jets observed with colour flow Doppler on the valves and the treatment. A chi-squared test was also performed to verify the presence/absence of arrhythmias among the three groups in relation to the treatment.

The Kruskal-Wallis test and Dunn’s multiple comparisons test were applied to verify differences in the number of arrhythmias diagnosed for each group.

The Friedman test was performed to compare the echocardiographic measurements obtained in WT, RT and RDT groups.

Statistical analysis was performed using commercial softwares (Anova and Friedman: JMP 16 –SAS, USA; chi-squared test: excel, Microsoft, USA; Kruskal-Wallis: GraphPad Prism 9, USA). Statistical significance was set at p < 0.05.

## 3. Results

One of the thirteen mares selected was excluded due to organization problems. The study protocol was thus carried out on twelve Standardbred mares aged between 5 and 9 years old (mean±standard deviation: 7±1.6 years) and with a body weight ranging between 441 and 586 kg (mean±standard deviation: 510±46.6 kg). The three echocardiographic examinations were performed on all the animals within the times established without any adverse reaction to either the drugs or physical restriction. All the mares were used to the room and the stock where the examination took place and, apart from the effect of sedation, no abnormal behaviour was recorded. Within the WT group, mares were calm and cooperative and within the RDT group, no excitement effect was produced by dobutamine infusion.

### 3.1. Monitoring

**Fig 2:**
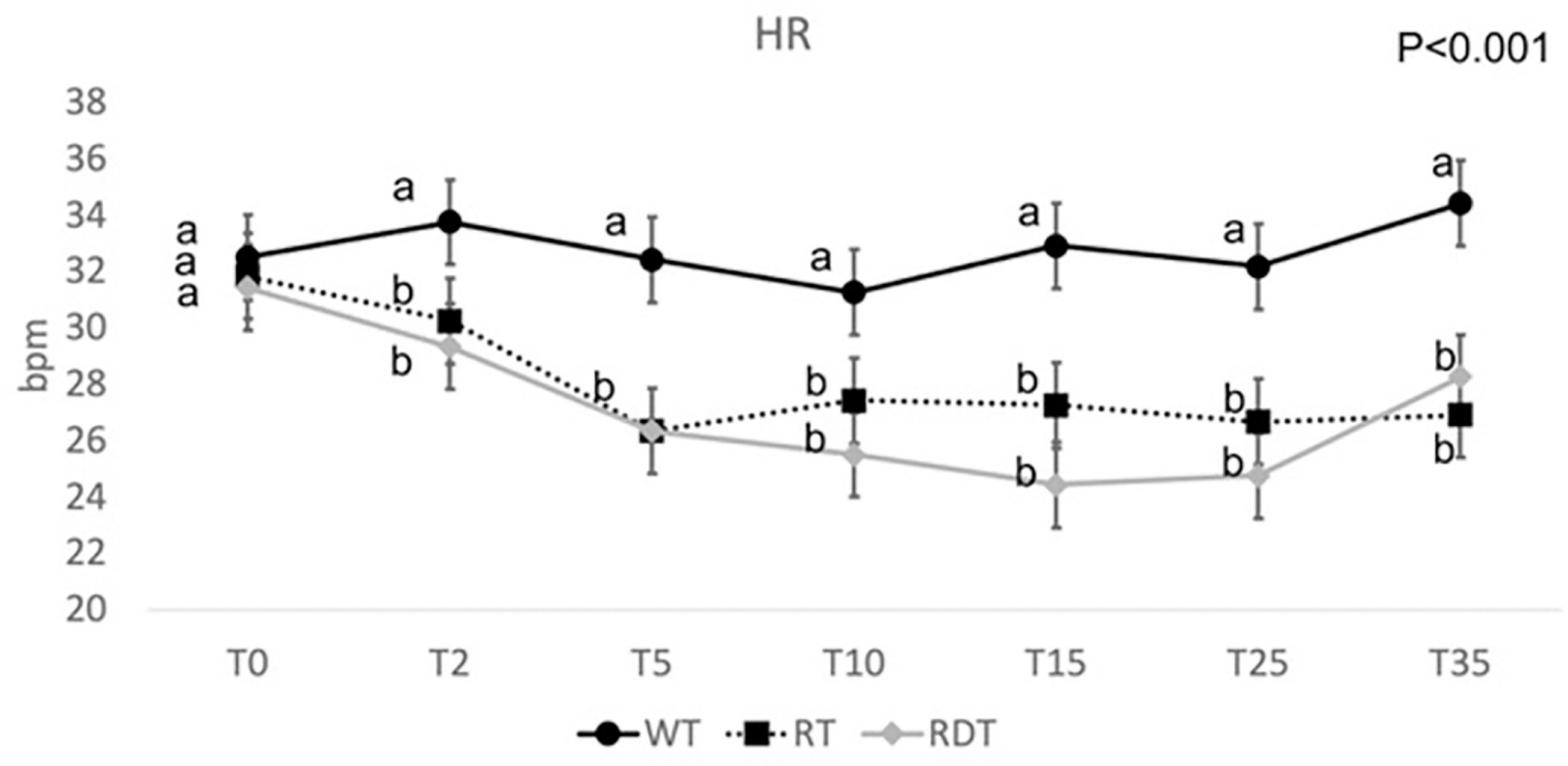
Mean heart rate (HR) and standard error at 0, 2, 5, 10, 15, 25 and 35 minutes for WT, RT and RDT groups. WT = without treatment; RT = romifidine treatment; RDT = romifidine and dobutamine treatment. The different letter indicates a significantly different value between groups at the time points.

Mean HR recorded during the procedure was significatively higher in the WT group [mean +/- standard error 32 (30–38) bpm] compared with the RT [28.5 (19–36) bpm] and the RDT [26.5 (21–42) bpm] groups (Fig 2).

Regarding the presence/absence of arrhythmias, differences were found between WT and RT (p = 0.0444) and RDT (p = 0.0444) groups, but no differences were found between RT vs RDT.

Also, in terms of the number of arrhythmias, differences were found between WT and the other two groups (both p<0.0001). In the WT group, most of the arrhythmias were Mobitz type II second-degree atrio-ventricular blocks (II-AVB), but one mare also presented sinus blocks in which the RR intervals were equal to 2 normal RR intervals (SB). In the RT group, and in the RDT group, 3 and 5 additional mares also presented SB. In addition, in the RDT group, two mares showed atrial premature contractions, characterized by a premature P followed by a QRS with normal morphology and duration (APC) and two isolated ventricular premature contractions, characterized by an abnormal QRS not preceded by a P wave (VPC). Table 1 shows the number and the types of arrhythmias recorded in each group.

**Table 1:** Number of mares that presented arrhythmias in each group of treatment and the types of arrhythmias recorded. For each arrhythmia recorded during the whole 35-min period median and range values are reported within parentheses (median; range).

Treatment	Number of mares with arrhythmias	Number of mares with each type of arrhythmias			
		II-AVB	SB	APC	VPC
WT	7/12	7/12 (0; 0–173)	1/12 (0; 0–11)	0/12 (0; 0–0)	0/12 (0; 0–0)
RT	12/12	12/12 (210; 60–425)	4/12 (0; 0–9)	0/12 (0; 0–0)	0/12 (0; 0–0)
RDT	12/12	12/12 (301; 41–626)	5/12 (1; 0–92)	2/12 (0; 0–20)	2/12 (0; 0–13)

WT = without treatment; RT = Romifidine treatment; RDT = Romifidine and Dobutamine treatment; II-AVB = II-degree atrioventricular block; SB = sinus block; APC = atrial premature contraction; VPC = ventricular premature contraction.

**Fig 3:**
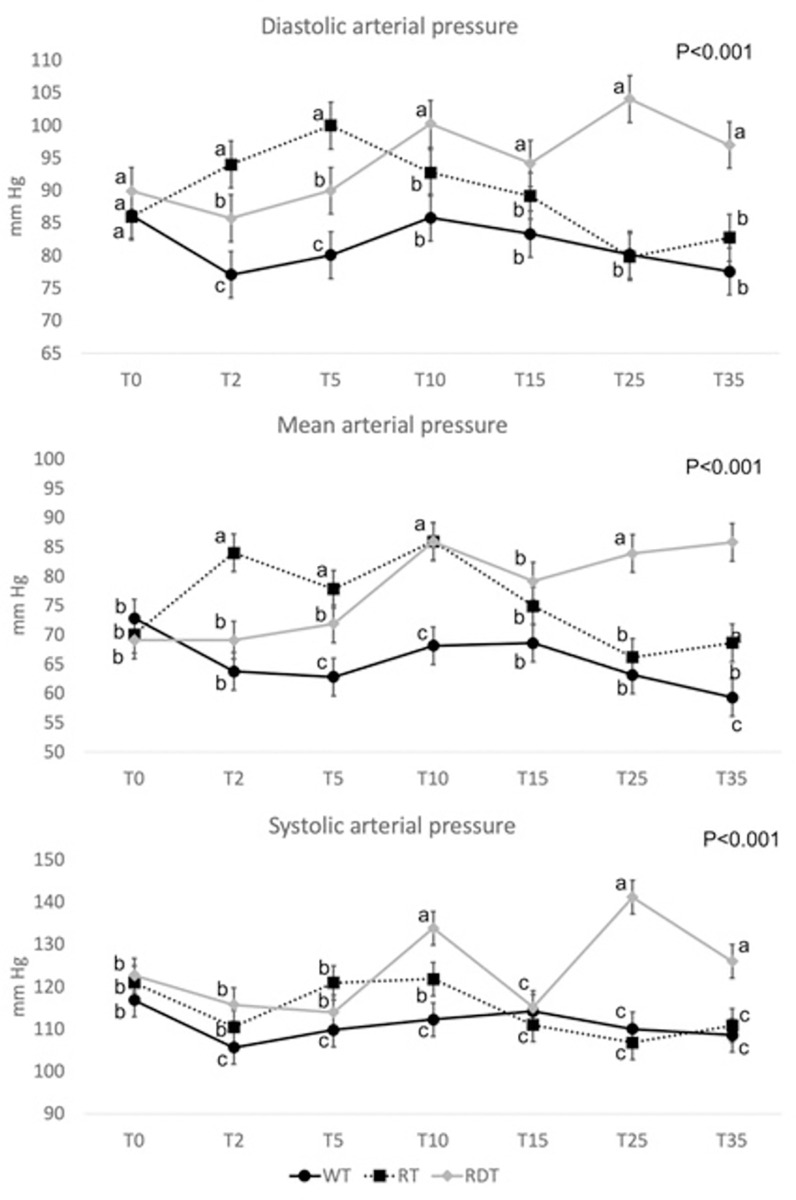
Average IBP and standard error at 0, 2, 5, 10, 15, 25 and 35 minutes for WT, RT and RDT groups. WT = without treatment; RT = romifidine treatment; RDT = romifidine and dobutamine treatment. The different letter indicates a significantly different value between groups at the time points.

In 34% of the set times, the NIBP system failed to record any value thus no statistical analysis could be performed; while IBP recordings were obtained in 10/12 mares, because due to behavioural issues, it was not possible to insert the intra-arterial catheter in two of the mares. Furthermore, in 3/10 mares no values were obtained during one of the three treatments due to the catheter becoming dislodged. In the remaining mares, in 13% of the set times, the system failed to record any value due to technical errors (zero set request or flushing needed). The IBP recorded was higher in the RDT group compared with the other two groups. Detailed values for each group are reported in Fig 3 as average values and standard deviation at each time point.

### 3.2. Echocardiography

Echocardiographic measurements obtained in the three groups of treatment are reported in Table 2 as the median with maximum and minimum values. LVID in diastole and LVFW in systole were increased in the WT group compared with the RT and RDT groups. FS and EF calculated from the LV volumes estimated with the Teicholz formula were also higher in the WT group compared with the RT and RDT groups.

**Table 2:** Echocardiographic measurements obtained in M-mode and B-mode for the three groups expressed as a median (minimum-maximum value).

	WT group	RT group	RDT group	P values
**M-mode measurements**				
IVS_d_ (mm) Normal range: 26.9–35.1	28.4 (21–32.5)	27.5 (20.2–34.8)	28.1 (22.6–34.4)	0.51
LVID_d_ (mm) Normal range: 103.1–128.9	103.2 (90.2–124)^a^	100.9 (87–110.2)^ab^	96.5 (84.5–115)^b^	0.008
LVFW_d_ (mm) Normal range: 24.9–25.5	26.2 (20–27.3)	24.2 (22–28.6)	24 (20.2–29)	0.368
IVS_s_ (mm) Normal range: 41.2–48.4	44.2 (36.5–52.5)	42.2 (37.4–47.7)	45.2 (37.2–52.6)	0.273
LVID_s_ (mm) Normal range: 63.7–84.7	60.1 (48.6–76.9)	64.1 (49.3–75.1)	60.8 (53.5–73)	0.097
LVFW_s_ (mm) Normal range: 31.2–41.6	44.9 (37.5–58.1)^a^	39.7 (34.4–47.6)^b^	39 (35.8–44.9)^b^	0.005
FS (%) Normal range: 32.3–40.1	41.5 (36.6–52)^a^	36.3 (30.2–43.2)^b^	37.2 (28.4–43.8)^b^	0.001
**B-mode measurements**				
LV area_d_ (cm^2^)	128.5 (105–165.9)	130.1 (108.3–146.2)	132.8 (115.8–194)	0.173
LV area_s_ (cm^2^)	60.6 (44.5–72.7)	58.7 (51.8–74.8)	60.8 (51.4–73.4)	0.212
EF (%)	68.9 (61.4–79.2)	66.1 (59.8–73.2)	69.9 (63.6–75.4)	0.075
SV (ml)	599.8 (434.5–816.8)	571.8 (486.3–784.9)	648.9 (491.7–966.5)	0.098
LA (mm) Normal range: 109–119	118.9 (112–125.8)	121.1 (108.5–127.5)	118.9 (109.5–132.5)	0.559
AO (mm) Normal range: 75.3–81.9	77 (69.4–83.9)	78.1 (69–87.8)	78.2 (67–88.1)	0.075
PA (mm) Normal range: 50.3–57.9	57.8 (46.4–62.6)	54.9 (46.6–58.5)	55.8 (54.3–71.1)	0.558

IVS_d_ = Interventricular septum in diastole; LVID_d_ = Left ventricular internal diameter in diastole; LVFW_d_ = Left ventricular free wall in diastole; IVS_s_ = Interventricular septum in systole; LVID_s_ = Left ventricular internal diameter in systole; LVFW_s_ = Left ventricular free wall in systole; FS = Fractional shortening; EF = Ejection fraction; LV area_d_ = Left ventricular area in diastole; LV area_s_ = Left ventricular area in systole; LA = Left atrium; AO = aorta; PA = pulmonary artery; WT = without treatment; RT = romifidine treatment; RDT = romifidine and dobutamine treatment.

Within a row, values with different superscript letters differ significantly (P < 0.05).

Reference ranges from: Zucca E et al. 2008 [21].

No differences were found in the other M-mode values or in the measurements of LV area both in systole and in diastole, LA, AO, PA, stroke volume (SV), and EF calculated in B-mode.

In the WT group, small regurgitant jets on the tricuspid valve, very small and small regurgitant jets on the aortic valve, and very small and small regurgitant jets on the pulmonary valve were observed in 1, 4 and 8 mares, respectively. In the RT group, small regurgitant jets on the tricuspid valve, very small and small regurgitant jets on the aortic valve, and very small and small regurgitant jets on the pulmonary valve were observed in 2, 9 and 10 mares. Finally, in the RDT group, small regurgitant jets on the tricuspid valve, very small and small regurgitant jets on the aortic valve, and very small and small regurgitant jets on the pulmonary valve were observed in 2, 11, and 8 mares. One mare in the RDT group also presented a mild regurgitant jet on the pulmonary valve.

No regurgitant jets were found in any of the horses in any of the groups for the mitral valve. The incidence of regurgitant jets observed was not different between groups for tricuspid and pulmonary valves, however, it was higher in the RDT group compared with the other two groups for the aortic valve (p = 0.043).

## 4. Discussion

The present study describes echocardiographic measurements in a group of 12 healthy mares that were subjected to three randomized examinations: without any treatment, with romifidine sedation, and with romifidine sedation associated with a continuous rate infusion of dobutamine.

The HR of the mares was significantly lower under sedation, both with and without the use of dobutamine, compared with no treatment. Similarly, as expected from a previous study [22], a significantly higher frequency of arrhythmias was observed when romifidine, alone or with dobutamine, was administered. Although the image evaluation and measurements were performed “blinded”, because of the pronounced treatment effect on the HR, the WT group was easily recognizable from the other two.

As previously described [18,19], similar BP values were obtained with the NIBP and IBP system in the WT group. In the RT and RDT groups, the oscillometric NIBP device failed to reliably display the measurements due to the frequent II-AVB. However, the aim of the study was not to compare the two devices, as they were used simultaneously, but just to have at least one measurement per set time as an indication of the vascular effects of the drugs administered. In the RT group a transient rise in blood pressure was recorded from 2 to 10–15 minutes after the administration, while dobutamine infusion caused a sustained increase in SAP, MAP and DAP compared with WT group.

Regarding the echocardiographic measurements, several parameters, such as aortic diameter (p = 0.075), pulmonary diameter (p = 0.558) and left atrium diameter (p = 0.559) showed no differences between the three examinations.

Contrary to what has been previously described [4,5], we found no significant difference in LVIDs or IVSs between the three groups. Moreover, LVIDd was reduced in the RT and RDT groups compared with WT. This is in contrast with previous studies, where it was found to increase as low HR increases the diastolic filling time and thus the size of LVIDd [4]. However, this difference in the diastolic dimensions of the LV found in M-mode was not confirmed by the bidimensional assessment of the LV. In fact, the diastolic area of the LV in B-mode was found to have increased, although not statistically significant, in the RT and RDT groups compared with the WT group.

The possible disagreement between the M-mode and B-mode in the assessment of LV size could be due to the timing in which the images were obtained. In fact, there were 10–25 minutes of delay between the M-mode and the B-mode measurement, thus, it is possible that at the beginning of the ultrasonography the vasoconstrictive effect of romifidine and the combination of romifidine and dobutamine predominated over the bradycardia, while 10–25 minutes later the bradycardia led to the typical dilation of the LV in diastole reported by others [4]. This is supported by the trend of HR and IBP showed in Figs 2 and 3. Indeed, in the RT group, HR decreased significatively immediately after injection and remained low at all time points; while IBP increased significantly during the first 10 minutes but then returned similar to the WT group. On the other hand, in the RDT group, HR remained low and IBP high at all time points.

In agreement with previous studies [4,5], LVFWs significantly decreased with the alpha-2 agonist, regardless of the dobutamine infusion, indicating a reduction in systolic LV performance. Consequently, FS was also lower in the RT and RDT groups than in the WT group. These changes indicate a reduction in LV contraction which could be attributed to both the negative inotropic effect of romifidine and an increase of the afterload that can be caused by both sedation and the dobutamine infusion [9,23,24]. The alpha-adrenergic effect of both drugs results in peripheral vasoconstriction which increases the afterload. The direct beta1-mediated positive inotropic effect of dobutamine was thus probably insufficient to improve the LV contraction. It is possible that a higher dose of dobutamine is needed to counteract the negative inotropism of the sedation, although the concomitant effect on peripheral vascular resistance, may render this difficult to achieve.

As already reported, alpha-2 agonists tend to increase the incidence of regurgitant jets at the semilunar valves because of the prolonged diastole and increased afterload [4]. In our study, the occurrence of aortic regurgitation was even higher in the RDT group, possibly due to the summation of the alpha-adrenergic effect of both drugs on peripheral vascular resistance with an increase in the afterload.

The study has some limitations. First, as we were unable to include the minimum number of horses required (12 *vs*. 13), some differences might have not been highlighted. Moreover, due to the reduced number of subjects, it was not possible to perform a statistical comparison to verify whether the differences observed were imputable to the treatment group or the treatment order, thus we cannot exclude a summation effect of romifidine when it was administered on two consecutive sessions. However, although in a previous study [16] a reduced heart rate was found up to 300 minutes following romifidine administration, we observed a significative different heart rate in the WT group compared with the other two in which romifidine was used. Furthermore, to the 3 hours wash-out period, we have to add the 35 minutes in which the monitoring and ultrasound took place, thus the shortest time between two consecutive sedations would have been of 215 minutes. Another limitation is that the echocardiographic images were obtained only from the right side and there are some measurements which are best evaluated from the left side (i.e. left atrium size, mitral regurgitation). As the animals were familiar with the procedure before the initiation of the study, they were very calm and needed no restraining.

The lack of sympathetic stimulation during the baseline echocardiography may be responsible for the small differences observed in the examinations performed under sedation. These results are therefore not extrapolatable to horses that might require sedation due to their temperament, and thus are expected to have a high sympathetic tone. All the mares used were untrained and therefore, again, the results may not be extendable to performance horses.

Another limitation of this study is that troponin I was not measured prior to and after the use of dobutamine to assess the development of myocardial damage. It has however been measured in previous studies with higher doses of dobutamine and the level was never found to be above the reference values [14,25,26], thus cardiac myofiber injury with our dosage was considered unlikely. Moreover, in this study all the animals included were healthy, thus the effect of dobutamine in horses with cardiac problems is unknown. Finally, although the measurements were performed offline and intended to be “blinded”, the video loops recorded under the effect of sedation were easily recognizable due to the bradycardia and the II-AVB.

In conclusion, continuous rate infusion of dobutamine at the dose of 2 mcg/kg/min does not enable the alterations caused by sedation to be corrected and led to more similar measurements to those obtained after romifidine administration, than without treatment. It is possible that the dobutamine dose used in this study was too low, however, changes in the hemodynamics due to the two drugs might not allow physiological ventricular contraction and function to be restored.
